# Stabilizing and Destabilizing Factors of Nurse Retention: A Comparative Analysis of Two Healthcare Settings in Austria

**DOI:** 10.1155/jonm/8957256

**Published:** 2026-08-07

**Authors:** Tanja Lesnik, Olivia Kada

**Affiliations:** ^1^ University of Klagenfurt, Klagenfurt am Wörthersee, Carinthia, Austria, uni-klu.ac.at; ^2^ Carinthia University of Applied Sciences, Feldkirchen in Kärnten, Carinthia, Austria

**Keywords:** critical care, long-term-care, nurse, qualitative, retention, turnover

## Abstract

**Background:**

The global nursing shortage poses a significant threat to healthcare systems. Despite the extensive corpus of research gathered over several decades, there remains a notable gap in the comprehensive understanding of the underlying factors influencing nurses’ retention decisions, particularly in critical care (ICU) and long‐term‐care (LTC) facilities.

**Aim:**

This study aims to identify and analyze the nuanced differences and similarities in stabilizing and destabilizing factors that impact frontline nurses’ decisions to remain in or depart from critical care and LTC facilities.

**Methods:**

To achieve this objective, semistructured interviews and focus group discussions were conducted with 52 participants, including nursing managers and frontline nurses from nine hospitals and eight nursing homes in Austria. Data were analyzed using qualitative content analysis.

**Findings:**

The findings revealed several on‐the‐job stabilizing factors, including empowering leadership, a positive team culture, and work autonomy. Off‐the‐job stabilizing factors encompassed family time, proximity to the workplace, and professional prestige, the last of these being particularly salient for critical care nurses. On‐the‐job destabilizing factors included disproportionate individual responsibility, ethical challenges, and power dynamics, with slight variations between the two settings. Off‐the‐job destabilizing factors comprised caring responsibilities for relatives, professional advancement opportunities elsewhere, and the ease of movement within the nursing labor market.

**Discussion and Conclusion:**

Notable distinctions emerged between the two settings: Critical care nurses emphasized collaborative team culture and ethical dilemmas tied to pronounced hierarchical structures as central concerns, whereas LTC nurses were more affected by limited physician availability, feelings of being undervalued, and restricted opportunities for professional advancement. Overall, while several stabilizing and destabilizing forces were shared across both settings, their salience and expression differed meaningfully, underscoring the need for context‐specific retention strategies tailored to the distinct challenges faced by critical care and LTC nurses.

## 1. Introduction

The nursing workforce shortage represents a long‐standing issue that poses a substantial global threat to healthcare systems. The COVID‐19 pandemic has significantly exacerbated this crisis, leading to a critical juncture in the provision of healthcare services [1, 2]. The WHO [2] forecasted a shortage of 5.9 million nurses in the world’s healthcare systems by the end of 2030. Although Austria has not yet encountered a critical shortage of skilled workers to the extent observed in certain other OECD nations, such as Slovenia, Greece, Latvia, and Estonia [3], it is already facing significant challenges in the recruitment and particularly in the retention of nursing professionals across various settings. According to the Austrian Ministry of Health, registered nurses are one of the nationwide shortage occupations and 58,000 nurses will be needed only in the hospital sector in Austria by 2030 [4]. Studies show that the average career span of a nurse in the healthcare sector is only 6–10 years before they decide to leave the profession entirely [5]. Particularly in high‐pressure settings, like the critical care sector (intensive care unit [ICU]) [6] or settings that are already particularly affected by a lack of qualified nursing staff, such as the long‐term‐care (LTC) sector [7], the problem of voluntary turnover is omnipresent. High workload, psychological stress, ethical challenges, high mortality rate of patients, and increasing problems with adequate staffing levels exacerbate the problem of voluntary departure of nurses in these specialized settings [6–11]. After decades of research on voluntary turnover [12, 13] and extensive investigations into the factors that stabilize or destabilize nurse retention, comparisons of different contexts have been neglected.

Research indicates that job satisfaction [14] or sufficient staffing levels [15] and empowerment of nursing management [8, 16] play crucial roles in influencing nurses’ decisions to continue or quit their positions. Lee and colleagues [17] contend that numerous professionals, despite experiencing low job satisfaction, remain in their positions, while others choose to depart even when their job satisfaction is high. These observations indicate that job satisfaction alone cannot fully explain the dynamic interplays of voluntary turnover decisions.

Therefore, our objective is to identify and analyze the nuanced differences and similarities in perceived stabilizing and destabilizing factors that influence retention decisions among critical care and LTC nurses in publicly owned healthcare institutions in Austria. This study is guided by the following research questions:

• What are the key on‐the‐job and off‐the‐job stabilizing and destabilizing factors that affect critical care and LTC nurses’ decisions to remain in their work environments?
• How do these factors vary across distinct settings, and what are the underlying similarities and differences?
• Are stabilizing and destabilizing factors perceived equally between nursing managers and frontline nurses?

The findings of this study will hold direct significance for nursing management practice. By identifying context‐specific stabilizing and destabilizing factors in two distinct healthcare settings, care managers in ICU and LTC facilities are equipped with an empirical basis for designing and implementing targeted retention strategies. Understanding which factors promote or hinder nurse retention in each setting enables nursing managers to prioritize interventions, whether organizational, interpersonal, or structural, that are most likely to be effective in their specific context. This is particularly relevant given the growing nursing shortage in Austria and the need for evidence‐based workforce management approaches.

### 1.1. Theoretical Background and Literature Review

Mitchell and colleagues’ [18] job embeddedness (JE) theory was selected as the guiding framework for this study for three interconnected reasons. First, unlike earlier theoretical models that focus primarily on job satisfaction (1977) or organizational commitment [19] as predictors of turnover, JE shifts the analytical focus toward the forces that explain actual retention behavior (why nurses actively choose to stay) [17, 18]. This distinction is particularly relevant in nursing, where Lee and colleagues [17] have demonstrated that the relationship between satisfaction and staying decisions is far from straightforward. Some nurses remain in their positions despite low job satisfaction, while others depart even when satisfaction is high. JE accounts for this complexity by moving beyond attitudinal proxies toward a broader constellation of organizational, social, and community‐based forces that anchor nurses to their settings. Second, JE’s inherent dual structure, distinguishing between on‐the‐job and off‐the‐job dimensions, is uniquely suited to the comparative, context‐sensitive nature of the present study. Retention decisions in specialized nursing settings such as the ICU and LTC are shaped not only by workplace factors but also by forces originating in nurses′ private and community lives, such as family responsibilities, geographic proximity, and professional prestige. JE captures both domains within a single coherent framework, enabling a holistic analysis that purely organizational models cannot offer [20]. Third, JE directly informed the methodological design of this study: The on‐the‐job/off‐the‐job distinction guided the development of the interview guide, and the three JE dimensions, fit, links, and sacrifices, provided the deductive structure for the qualitative content analysis, ensuring theoretical consistency from data collection through to interpretation. The JE framework comprises three dimensions, fit, links, and sacrifices, each operating at both the community (off‐the‐job) and organizational (on‐the‐job) level. Fit describes the perceived compatibility between the individual and their work environment or community, links refer to formal or informal connections with colleagues, teams, or community members, and sacrifices capture the perceived costs of material or psychological loss associated with leaving one’s position [18].

Building on this theoretical foundation, the following section reviews the existing empirical research on nurse turnover and retention, in order to identify where the JE‐informed perspective of this study contributes to closing existing gaps in the literature.

There are a limited number of studies that have explored actual turnover decisions, especially from the perspective of nurses who have recently left their roles [21–23] or those who are considering staying in their work environment [23]. One literature string focuses on theoretical models related to job satisfaction and dissatisfaction, anticipating turnover [14, 24, 25], while a second string focuses on the impact of organizational commitment on turnover intentions [19, 26, 27]. Across both literature strings, the majority of studies focus on the general nursing population, with comparatively few examining specialized fields such as critical care or LTC [7, 28–30].

This focus on the general nursing population constitutes a critical gap, as the existing studies largely examine the intention to leave rather than the actual, lived determinants of staying decisions, which differ from the reasons for leaving [31]. Comparative investigations across specialized nursing settings remain largely absent from the literature. ICU and LTC represent two deviant settings, acute, high‐intensity care versus sustained, relationship‐based LTC, and the structural demands, patient populations, and professional cultures of these two settings differ substantially. Retention strategies effective in one context may therefore be ineffective or even counterproductive in the other [7, 11]. Furthermore, while Austria faces a growing nursing workforce crisis [4, 5], no study to date has empirically investigated the stabilizing and destabilizing factors shaping retention decisions across these two specialized care settings in the Austrian healthcare context. This is precisely the gap the present study aims to close, by examining, through a JE‐informed lens, why nurses in Austrian ICU and LTC settings choose to stay.

## 2. Data and Method

### 2.1. Study Design

A qualitative case study approach [32] was selected to gain a deeper understanding of the individual experiences of frontline nurses and nursing managers regarding the stabilizing and destabilizing factors in critical care and LTC facilities. Initial contact was made with nursing directors serving as gatekeepers, followed by snowball sampling techniques to finalize the sample. Semistructured interviews with nursing managers and frontline nurses, along with focus group discussions (see Table 1), were conducted to enhance this understanding. Two deviant and specialized settings were selected to investigate the differences and similarities of perceived stabilizing and destabilizing retention factors.

**TABLE 1:** Data inventory.

Data type	Context and quantity	Original data audience
Interviews	ICU: 16 interviews with 16 nursing managers; LTC: 10 interviews with 10 nursing managers; LTC: 10 interviews with 10 frontline nurses	Nursing managers of all levels (lower, middle, and superior management) and frontline LTC nurses

Focus group discussions	ICU: 4 focus group discussions with a total of 16 frontline nurses;	Frontline ICU nurses

### 2.2. Participants and Setting

This qualitative case study includes a total of 52 participants from two distinct public healthcare facilities in Austria: 32 (16 frontline nurses and 16 nursing managers) from critical care and 20 (10 frontline nurses and 10 nursing managers) from LTC (see Additional file 1). A purposive two‐stage sampling strategy (Additional file 1) was employed to identify and approach gatekeepers (chief of nurses across 10 public hospitals and nine public nursing homes) based on their roles and institutional contexts. Following initial contact with gatekeepers, snowball sampling techniques were used to recruit additional participants (nursing managers [*n* = 26] and frontline nurses [*n* = 26]) through the networks established by the gatekeepers, ensuring that a diverse and knowledgeable sample was reached.

We reached out to 19 institutions; however, one hospital and one nursing home declined our request due to insufficient staffing resources to participate in the study. In total, we included 17 institutions (nine hospitals and eight nursing homes) in the study. Consistent with the demographic distribution of the nursing workforce, a significant majority of the nursing managers (*n* = 20) and frontline nurses (*n* = 20) who took part in this study were female (see Table 2). All participants were informed about the study and provided informed consent before conducting the interviews.

**TABLE 2:** Characteristics of study participants.

	ICU		LTC	
	Nursing managers (NM)	Frontline nurses (FN)	Nursing managers (NM)	Frontline nurses (FN)
*Gender*				
Male	5	5	1	1
Female	11	11	9	9
Nonbinary	—	—	—	—

*Age*				
< 20	0	0	—	—
21–30	1	0	—	1
31–40	2	6	2	4
41–50	5	3	3	3
51–60	7	7	5	2
> 60	1	0	—	—

*Additional education*				
Yes	16	16	10	5
No	—	—	—	5

*Professional experience*				
< 1	2	0	—	—
2–5	6	0	1	3
6–10	4	4	3	1
11–15	2	3	3	2
16–20	1	0	2	1
> 20	1	9	1	3

*Level of nursing management*				
Ward manager	7	—	3	—
Nurse department manager	3	—	—	—
Chief of nurses	6	—	7	—

*Hospital type*				
Standard public hospital	3	0	—	—
Specialty public hospital	8	14	—	—
Central public hospital	5	2	—	—

*Nursing home type*				
Skilled nursing facility	—	—	10	10

### 2.3. Data Collection

Firstly, the study involved interviews with nursing managers and focus group discussions with frontline nurses in critical care settings, focusing on the stabilizing and destabilizing factors affecting their retention in these environments. Secondly, we interviewed nursing managers and frontline nurses from LTC facilities followed by the data triangulation of these two datasets. The aim of this comparative design is to obtain context‐specific insights pertaining to the phenomenon under investigation. Data collection took place between December 2023 and April 2024. Semistructured interviews were conducted in person, via Zoom, or by phone, while focus group discussions took place in person. All information of both interviews and focus group discussions was audio‐recorded. During the interviews, additional notes were made as deemed appropriate to capture pertinent information. Interviews with critical care nursing managers ranged from 21 to 87 min in length. Focus group discussions with three to five critical care frontline nurses per group lasted between 130 and 167 min. Interviews with LTC nursing managers varied from 18 to 64 min, and those with LTC frontline nurses ranged from a minimum of 20 min to a maximum of 40 min.

The interview guidelines (Additional file 3) comprised about 11 questions, which include questions like the following:

• What is the main reason why you feel comfortable in your field as a nurse and want to continue working there?
• Do you feel that you can realize your potential in your current area and make good use of your strengths?
• In your experience, what are the reasons why qualified nursing staff have left the intensive care sector or LTC sector voluntarily?

The first author, who is experienced in the relevant contexts and qualitative data techniques, created the interview guide and tested it for clarity among frontline nurses before use. Subsequently, both authors trained three healthcare management students to assist with additional data collection. Thus, data collection has been conducted by the first author and the students.

### 2.4. Data Analysis

All focus group discussions and interviews were recorded and transcribed verbatim to ensure anonymity. The resulting transcripts were subjected to qualitative content analysis according to Mayring [33]. This approach facilitated a systematic examination of the data, allowing for the extraction of meaningful themes and insights. This structured method encompasses both inductive and deductive categorization processes to thoroughly analyze the phenomenon. The raw data were segmented into suitably small units of content, thereby facilitating a detailed investigation.

Deductive techniques were used to structure the material according to the theoretically relevant dimensions (on and off the job stabilizing and destabilizing factors) derived from the framework of JE [18, 20]. In the next step, subcategories were developed inductively for each deductive main category. First, part of the material (60%) was coded parallelly by both authors. Categories were then compared and refined, and the final set of categories was defined including category definitions, coding and rules, and example quotations to support intersubjective comprehensibility [33]. Then, the remaining material was split and analyzed. After coding 50% of the data, the authors engaged in peer debriefing as a logical form of reliability check [34]. This collaborative debriefing was repeated upon completing the independent coding to address any potential misinterpretations. The entire process was documented to ensure comprehensibility and trustworthiness, and data analysis was supported by MAXQDA software.

### 2.5. Credibility and Trustworthiness

The study was reported in accordance with the Consolidated Criteria for Reporting Qualitative Research (COREQ) checklist [35] (Additional file 4) and Guba and Lincoln’s [36] principles (Additional file 5) were applied to ensure credibility and trustworthiness. To maintain credibility throughout the study, we collected interview data from various knowledgeable actors and dedicated 5 months to data collection. When analyzing the data, we remained cognizant of potential biases and utilized a peer debriefing strategy to enhance confirmability.

### 2.6. Ethics

This study was approved by the ethical committee of the University of Klagenfurt (Ethical Approval No.: 2023‐053). Informed consent was obtained from all study participants and institutions the participants work in. The researchers explained the aim and the approach of the study to the participants and received the written consent before the interviews. Interviewees have been deidentified, and the study ensured anonymity and integrity of the data. Data thus include personal information and cannot be disseminated to the public.

## 3. Results

### 3.1. Characteristics of the Sample

A total of 52 participants (see Table 2) were interviewed about their perceived stabilizing and destabilizing factors regarding nurse retention in critical and LTC facilities. Seventy‐seven percent (*n* = 40) of the interviewed participants were female and 23%, (*n* = 12) were male. Over half of the participants (61.5%) were critical care frontline nurses and nursing managers, whereas 38,5% belonged to LTC facilities. The majority of the nursing managers were between 51 and 60 years old, and their professional experience ranged from one to over 20 years. 56.2% of ICU nurses had over 20 years of professional experience, and all of the interviewed frontline nurses had additional education (ICU training, nursing science studies, dialysis training, anesthesia training, etc.), whereas only half of the LTC frontline nurses had additional education, and only 30% had more than 20 years of professional experience.

### 3.2. Main Categories and Subcategories

The findings (coding guideline is illustrated in Additional file 2) are presented along two main categories derived from the literature [17, 18]: (1) on‐the‐job and (2) off‐the‐job stabilizing and destabilizing factors in critical and LTC.

### 3.3. On‐the‐Job Stabilizing Factors

Stabilizing factors in the organization (on‐the‐job) refer to the various factors that arise from the working environment of nurses and promote retention in their setting.

*Empowering Leadership and Positive Team Culture (n = 46)*. Interviewees (both managers and frontline staff) in both settings mentioned an empowering leadership (LTC: *n* = 15, ICU: *n* = 22) and a positive team culture (LTC: *n* = 14, ICU: *n* = 31) as the key factors for keeping nurses in their settings. Nursing managers of the ICU (*n* = 10) pointed out that a supportive and empowering leadership culture with an open error management is essential for retaining their workforce.

They mentioned that nursing managers in every level are responsible for creating a collaborative and relational team culture, but particularly, it is the role of the ward manager to invest in these processes.

Also, in the LTC setting (*n* = 15), a positive team culture was one of the most important stabilizing forces for retention for both frontline nurses and nursing managers.

“I do think that the manager should take time for the employees and that everyone is listened to and talked to and asked how they are doing and that the employee simply feels comfortable and at ease in the team and that they don’t have to be afraid if they ask the management something. That they can simply talk about it openly. The feeling of well‐being and communication and valuable interaction. (ICU_nurse_4, Pos. 346)”

Frontline nurses of both settings (LTC: *n* = 6, ICU: *n* = 12) mentioned that they value supportive, participative, and caring nursing managers. However, critical care nurses and their interviewed nursing managers pointed to the fact that within critical care, one is forced to practice good collaboration within the team, whereas in other settings, you can more easily carry out some nursing tasks individually. Although the positive team culture and support of colleagues was important for frontline nurses in both settings (LTC: *n* = 9, ICU: *n* = 16), it was essential and more often discussed for nurses in critical care (*n* = 16). They mentioned that the reason for staying in the setting is their interdisciplinary team colleagues.

*Organizational Conditions and Work Environment (n = 175)*. Work autonomy (LTC: *n* = 6, ICU: *n* = 11) and high quality of delivered care (LTC: *n* = 9, ICU: *n* = 16) seem to represent significant contributors in both LTC and critical care facilities. Frontline nurses in both settings would rather leave the setting than delivering low quality of healthcare to their patients. They emphasize that the patient or resident is at the center and deserves to be treated in a professional way with high‐quality health services.

“The diverse care at the intensive care unit. All the things we’re allowed to do autonomously, organise ourselves and take responsibility for the work we do. Care is at the top of my list. I love to see how it helps the patient. If someone is allowed to die then I can also be there and accompany them, that’s how it should be, and with others we make sure that the patient progresses positively. The patients and caring for them professionally and well is crucial. (Focus group__ICU_nurse_4, Pos. 207)”

Nursing managers mentioned that giving a voice to their employees (LTC: *n* = 9, ICU: *n* = 16) and creating professional freedom to deliver high‐quality care are essential components for long‐term retention. Besides that, the enrollment period, and transition to the setting, plays a major role. Nurses in critical care (*n* = 12) reported that positive experiences and support during their enrollment period contributed positively to their decision to stay in the setting. Nursing managers (*n* = 3) explained that structured enrollment processes with sufficient knowledge provision and support in challenging situations are cornerstones for remaining the nursing workforce in critical care. Additionally, providing nurses with all the necessary further training (LTC: *n* = 17, ICU: *n* = 14), they need endows them with important resources. Age‐and‐family‐friendly shifts are one of the most important contributing factors for retention in both setting (LTC; *n* = 9, ICU: *n* = 22). Thus, nurses (LTC: *n* = 3, ICU: *n* = 8) and nursing managers (LTC: *n* = 6, ICU: *n* = 14) in critical care and LTC facilities argue that age‐friendly shifts and the deployment of resources of older employees will be essential parts of retention strategies in the future of securing the nursing workforce.

“Yes, and for the older generations, of which I am one, I would like us to be able to take advantage of programmes such as partial retirement when we get older. Because I have to be honest, you realise that after a certain time you’re no longer as productive as you used to be. If this is encouraged, there will also be fewer sick days, and more satisfaction. And perhaps older people can work longer and still do well in a company. That’s what I want. I would also like to be able to continue working there. (Interview 9_nurse_LTC_1, Pos. 9)”

*Remuneration and Social Benefits (n = 106)*. An increase in remuneration (LTC: *n* = 9, ICU: *n* = 28) was mentioned by all participants while only the nurses and their managers of LTC (*n* = 9) reported financial incentives for extra shifts. This difference likely reflects the fact that intramural (hospital‐based) settings more commonly offer financial incentives for additional shifts, whereas extramural (LTC) settings typically do not provide comparable compensation. Nursing managers in both settings (LTC: *n* = 4, ICU: *n* = 3) were nonetheless convinced that, compared to other healthcare professions, nursing remains underpaid. Several participants argued that equal pay should be established across all healthcare sectors, as facilities would otherwise compete with one another for nurses. Although frontline nurses in both settings (LTC: *n* = 4, ICU: *n* = 16) described salary as a determining factor, they considered empowering leadership, positive team culture, and supportive organizational conditions and work environment to be more crucial for remaining in their setting.

“Yes, I have to say that in the beginning, being a nurse was very attractive from a financial point of view. But I think it’s not a vocation anyway, so you can’t just do it for the money. Therefore, money is not the best motivator. (Interview 4_nurse_LTC_4, Pos. 1)”

The interviewees of both settings (LTC: *n* = 13, ICU: *n* = 20) also appreciated social benefits like health‐promoting offers, team building interventions, or lunch at the ward and free parking spaces in front of their institutions.

“Yes, a masseur comes to our institution, I think that’s pretty cool to take advantage of. It’s not free, but it’s cheaper, and yes, I think it’s great. We get food for a whole day. We pay 60 euros a month and get breakfast, lunch, a cake in the afternoon, dinner, there’s enough of everything. That has really halved my entire food costs in one month, which is really cool. Yes, exactly, all these little treats. (Interview 1_nurse_LTC_1, Pos. 7)”

### 3.4. Off‐the‐Job Stabilizing Factors

Stabilizing forces within the community (off‐the‐job) are factors that emerge out of the private environment and strengthen nurses’ retention.

*Time for Family and Proximity to Workplace (n = 41)*. Interviewees in both settings (LTC: *n* = 14, ICU: *n* = 24) emphasized that scheduling family and work (especially working part time) and having enough time for family stabilizes their decision to remain in their setting.

“I believe that it is becoming increasingly important for them to reconcile work and family life. I know this because we have just completed a project on this topic and conducted a survey (Interview 4_NM_ICU, Pos. 7)”

Frontline nurses in LTC facilities (*n* = 3) also consider the close distance of their institution to their homeplace as stabilizing because that adds valuable time to their family life.

“One of the main reasons why I still like working in the nursing home where I am now is that it’s really close to my home. So, I don’t need a car, I don’t have to drive to work for ages, I can be back home with the children quickly, and that’s another point that I actually really like, and that’s why I really enjoy working there. (Interview 3_nurse_LTC_3, Pos. 1)”

*Prestige (n = 4)*. Prestige was perceived as a stabilizing force only among frontline nurses in the critical care setting (*n* = 4). These nurses told us that they aimed for working in critical care and enjoy working there also because of the prestige in society. This gives them a special feeling and pride that they are the ones who deliver high‐quality care under special conditions.

“One of the reasons why I like working in intensive care and want to stay there is the ‘reputation’ or that it is a profession or job that you can be proud of and, above all, that it is very highly regarded in society. When you say that you are an intensive care nurse, everyone is always amazed. (Focus group_nurse__ICU_1_2, Pos.31)”

### 3.5. On‐the‐Job Destabilizing Factors

Destabilizing forces within the workplace (on‐the‐job) refer to the various factors that contribute to the voluntary leaving decision among frontline nurses.

*Responsibility and Ethical Challenges (n = 50)*. The concept of responsibility is intrinsically linked to work autonomy. Although work autonomy is perceived as a stabilizing factor for nurses in both settings, an overburdening of responsibility (LTC: *n* = 4, ICU: *n* = 1), particularly when carried solely by the individual, has been perceived as detrimental to job retention. This phenomenon was predominantly noted within the context of LTC settings (*n* = 4), where nurses frequently find themselves tasked with making critical decisions in the absence of physicians. The interviewees (LTC: *n* = 4, ICU: *n* = 1) expressed a desire for shared responsibility within an interdisciplinary team. They highlighted the absence of shared responsibility as a trigger for their leaving decision. As one interviewee pointed out:

“From my experience in the long‐term‐care sector, I think one main reason is the big responsibility that the qualified nurses in a nursing home have to bear and certainly the fact that we don’t have a doctor on site 24 hours a day. So, we don’t have a doctor in the institution around the clock, and we have a lot of responsibility and have to make a lot of decisions, so that’s certainly one of the main reasons, I would say, because we have a lot of responsibility and have to make a lot of decisions on ourselves. (Interview 5_NM_LTC, Pos. 1)”

In critical care settings, nurses (*n* = 8) and their superiors (*n* = 7) have reported that ethically challenging situations involving patients, such as moral distress and instances of missed nursing care, significantly impact their retention. Interviewees specifically emphasized the emotional turmoil caused by futile end‐of‐life care mandated by physicians and their inability to intervene. They also noted that organizational constraints and time limitations often hinder their ability to deliver high‐quality care, which makes them end in moral distressing situations. One of the interviewed nursing managers pointed to the fact:

“Then, I also believe that it is partly an ethical decision in the field of intensive care. We know that we are of course dependent on the doctors’ treatment decisions, that we also have to be involved when we care for a patient. And of course, there is not always a correlation between whether the therapy is limited or prolonged or ethically right. The nurses are always caught in a dilemma when they perceive the therapy as futile whether it is limited or extended. I think some of the nurses leave because of these ethical dilemmas. (Interview 7_NM_ICU, Pos. 1)”

Furthermore, interviewees (LTC: *n* = 12, ICU: *n* = 5) expressed concerns about being assigned numerous non‐nursing responsibilities, such as cleaning, answering phones, and performing tasks typically reserved for physicians or nursing assistants. This shifting of tasks has contributed to a significant reduction in the time available for patient care and is perceived as destabilizing in both areas, although nurses in intensive care (*n* = 5) emphasized this problem more strongly. In LTC settings, some frontline nurses (*n* = 10) are delegated to nursing aide duties, preventing them from fully utilizing their professional expertise as mentioned by the nurses and the managers.

“For some nurses, a key reason for their dissatisfaction is that when they are hired as nurses, they are not actually employed in that role. Instead, they often find themselves working as nursing aides or assistants. For instance, tasks such as changing dressings or managing permanent catheters are typically assigned to the ward manager. This leads them to feel frustrated, as they believe they are underutilizing their skills and competencies as fully trained nurses while working in the nursing home. (Interview I3_NM_LTC 3, Pos. 10)”

*Interpersonal Relations and Power Dynamics (n = 101)*. Nursing managers in both settings (LTC: *n* = 1, ICU: *n* = 4) noted that generational conflicts and interpersonal relations within teams play a significant role in destabilizing retention. Nurses also reported that these conflicts escalate into serious personal disputes (LTC: *n* = 4, ICU: *n* = 16) or even mobbing (LTC: *n* = 1; ICU: *n* = 1), prompting nurses to leave their positions. Additionally, many frontline nurses indicated that a dysfunctional team structure (LTC: *n* = 6, ICU: *n* = 16) is a key contributor in their decision to depart.

“In which case would I leave? If this team constellation gets completely out of hand and it’s no longer possible for me to work here. If there is bullying, if the boss is no longer behind me, if there is no open ear for the problems and needs of the employees. Or if the staffing situation gets even worse and I have the feeling that this is such an emergency situation that I can’t be part of it. I can no longer be a part of this team. (Interview 6_nurse_LTC_6, Pos. 7)”

Critical care nurses (*n* = 16) outlined the hierarchical dynamics within their teams and the broader hospital structure (*n* = 12) as destabilizing forces. Concerns have been expressed regarding the prevailing power dynamics that exist between nurses and physicians (*n* = 12), as well as between nurses and nursing managers (*n* = 12). These dynamics are perceived as destabilizing forces that significantly impact staff retention within critical care teams. While they noted a trend toward less hierarchical, interdisciplinary teams, problems related to abuse of power continue to drive nurses out of the profession.

“With doctors, it’s still partly the case that you have to obey, some are still like that. There are a few exceptions, but not as often as before. The hospital as a whole is very hierarchical, which is not necessarily a good thing. (Focus group_nurse_ICU_3_1, Pos. 201)”

*Staffing Resources and Shift Work (n = 39)*. Lack of staff was mainly reported by LTC nurses and their managers although staffing shortage was perceived in both settings (ICU: *n* = 3, LTC: *n* = 11). Interviewees from LTC mentioned that qualified staff is very hard to find, and sometimes, they have positions unoccupied for months, which shifts additional work to the nursing team. The staffing problem was also attributable to the constrained public budget in LTC facilities (*n* = 5) in Austria. Nursing managers of LTC settings (*n* = 5) stated that nurses in the hospital sector get paid higher salaries than in LTC. However, critical care nurses (*n* = 3) also mentioned that they experience staffing level problems; especially when there are sick leave or pregnancies; extra shifts have to be covered.

“A few years ago, they just ran out of staff in one residential area, there was a trainee and the cleaner who managed the whole ward and there wasn’t a qualified nurse or a proper nursing assistant on duty. There was just no other option, there was nobody else. That’s very problematic. (Interview 1_nurse_LTC_1, Pos. 6)”

Additionally, interviewees of both settings (LTC: *n* = 8, ICU: *n* = 7) mentioned that working in shifts and doing night shifts, or taking over extra shifts for several years is clearly negatively affecting their retention.

“Yes, exactly, then I still like the work. Because I know that on the other hand, I have my permanent job and my colleagues, who I still love. But then you realise that shift work is becoming more and more exhausting and I don’t like to do this anymore. (Focus group_nurse_ICU_2_2, Pos. 36)”

*Lack of Professional Challenge and Development (n = 11)*. LTC nurses and their managers (*n* = 5) identified a lack of professional challenge as a destabilizing factor in their work environment. Nurses noted that the inadequate utilization of their professional skills frequently prompts them to pursue employment in the hospital sector instead. Additionally, the limited opportunities for professional development (*n* = 6) in the LTC sector were also viewed as a destabilizing force, as highlighted by the following quote:

“There is no further professional development, there is nothing. Because the residents have to be cared for, how am I supposed to professionally develop further? There may be a course in aroma care that I can do, but we already have that, it’s standard. (Interview4_NM_LTC, Pos. 3)”

*Work-related Psychological Exhaustion and Trauma (n = 16)*. In critical care settings (*n* = 10) that accommodate critically ill patients and in LTC facilities (*n* = 2) with geriatric psychiatric patients, nurses frequently report experiencing considerable psychological exhaustion or trauma, prompting them to contemplate leaving their positions. Nursing managers in critical care (*n* = 1) and LTC (*n* = 3) have observed that some employees display pronounced signs of psychological trauma after years of enduring abnormal and traumatic situations.

“I have already said that the great danger of working in an intensive care unit for many years is emotional exhaustion or burnout. Unfortunately, emotional exhaustion is not that rare. This means that at some point the patient becomes a workpiece because I no longer have the opportunity to feel empathy. Because I’m simply too afraid of loss, afraid of failure, afraid of meeting the relatives again and seeing death or whatever. Or seeing the severe disability that comes out of all this. And that is one of the biggest challenges for me in the intensive care unit. Because emotionally exhausted employees don’t do the work because they want to, this is always a danger for everyone involved. And you can see that, you can see that in these alarm figures, how they deal with the patient and how they deal with the relatives. (Interview 11_NM_ICU, Pos. 37”

“Then we often have geriatric psychiatric cases where it’s not the body that needs to be treated, but the person’s own psyche. Then you really have to treat your own psyche, which is extremely challenging. (Interview 2_NM_LTC, Pos. 2)”

### 3.6. Off‐the‐Job Destabilizing Factors

There are various destabilizing forces within the private/community environment (off‐the‐job) contributing to voluntary turnover among frontline nurses.

*Caring for Relatives (n = 4)*. Care responsibilities of relatives were mentioned as retention destabilizing factors outside by frontline critical care nurses (*n* = 4). Employees start to reduce their working hours or decide to quit to dedicate themselves to the care of family members, as these interviewees explained.

“And we also have children or parents who need professional help because of an illness or whatever, so everyone has to take responsibility in their family too. (Focus group_nurse_ICU_3_2, Pos. 218)”

“When my mum fell ill, I myself doubted whether I would still be able to work in the ICU. So, there was always something that make me think I would have to change job. (Focus group_nurse_ICU_3_ 1, Pos. 25)”

*Professional Opportunities, Ease of Movement, and Image of Nursing Profession (n = 27)*. Many interviewees (LTC: *n* = 2, ICU: *n* = 5) expressed that the advancements in the nursing profession in Austria have greatly expanded their career opportunities. They noted that due to new professional fields like Community Nursing or Acute Community Nursing, their career opportunities have expanded and there are more willing to leave their workplace. Critical care nurses and their managers also emphasized that the current shortage of nurses (*n* = 11), and the resulting need for experienced nurses everywhere as well as new emerging professional fields are contributing to the ease of movement.

“For example, we now also have cases where staff have specifically moved away from intensive care, for example to the preclinical emergency field. We have a completely new profession elsewhere, this Acute Community Nurse (CAN), where intensive care staff suddenly switch to an extramural area. This is a particularly interesting project and didn’t exist in the past. Today nurses are more flexible. (Interview 11_NM_ICU, Pos. 13)”

In the field of LTC, participants (*n* = 8) claimed that the societal perception of this profession in Austria undermines staff retention, as working in LTC is often undervalued. It is commonly regarded as an inferior career choice. There is a desire for improved image campaigns to help attract and retain more nurses in this sector.

“I’ve actually always experienced it that way, because it always looks to the outside world as if you don’t need any specialist knowledge or expertise in the field of long‐term‐care, because you′re basically seen as auxiliary staff. So, you hear a lot of negative things, even within your own organisation. (Interview 7_nurse_LTC, Pos. 6)”

Additionally, the change of the severance payment scheme *“Abfertigung neu”* was perceived as destabilizing the retention of LTC nurses (*n* = 1) as well.

*COVID-19 Crisis (n = 6)*. Crises, such as COVID‐19, have been recognized as a significant challenge for long‐term (*n* = 3) and critical care (*n* = 3) nursing professionals. Reports indicate that during this period, some nurses in these settings faced heightened stress and increased workloads, leading to attrition among colleagues who left their positions as patient volumes surged. Interviewees also reported that the public healthcare system is not adequately resourced to tackle such crises.

“Yes, in recent years, especially after COVID‐19, it has actually become more and more difficult. Before, before COVID‐19, it was actually good to work, but during and after COVID‐19 I really have to say that there has been a rupture in some way, I think the workload in long‐term‐care is extremely high since then. (Interview 7_nurse_LTC_7, Pos. 1)”

## 4. Discussion

The findings of this study are broadly consistent with existing literature on nurse retention in general, while contributing novel context‐specific insights that extend prior work. Like Roth and colleagues [23], we identified empowering leadership, team culture, and work autonomy as central stabilizing factors. Similarly, to Tummers and colleagues [7], we found that staffing shortages and shift work destabilize retention, particularly in LTC settings. However, our study is distinct in several important ways. First, it applies a comparative case study design that explicitly contrasts ICU and LTC settings within the same national healthcare context, a methodological approach rarely employed in this field. Second, by integrating the JE framework with qualitative content analysis, we capture the nuanced interplay of on‐the‐job and off‐the‐job factors that most prior studies address only in isolation. Third, our sample includes both nursing managers and frontline nurses, offering dual perspectives that are frequently absent from the existing literature, which tends to focus on frontline staff alone.

*Empowering Leadership and Positive Team Culture* were identified as crucial on‐the‐job stabilizing factors in both settings. However, participants in ICU described a positive and collaborative team culture as essential, whereas LTC nurses only considered it important. Critical care nurses and their nursing managers argued that collaboration in ICU is more intense than on other wards and essential for patient safety. This aligns with other studies arguing that effective collaboration in healthcare teams decreases patient mortality rates [37], reduces adverse patient outcomes [38], increases the quality of delivered nursing care [39], and enhances patient safety [40]. These findings extend the prior literature by revealing a setting‐specific gradient. This nuanced distinction has not previously been reported in comparative nursing retention research and adds important setting‐specific insights.

*Organizational Conditions and Work Environments* seem to be another important retention stabilizer. Interviewees from both settings emphasized that, due to high work autonomy and being heard by nursing managers and other healthcare professionals, they are able to deliver the high‐quality care standards they aim to achieve. High‐quality care delivery thus has a stabilizing effect on frontline nurses across both settings, driven by nurses’ strong moral obligation to patient care. They also reported that a supportive and structured transition and enrollment period, incorporating knowledge‐sharing systems, career advice, and empowering decision‐making, contributed to their decision to stay in both ICU and LTC facilities. The centrality of work autonomy and high‐quality care delivery as stabilizing forces aligns with Kester and colleagues [15], who identified sufficient staffing and empowerment as key retention drivers. However, the current study adds important nuances. Nurses in both settings reported that missed nursing care due to organizational constraints actively triggered withdrawal behavior, a finding that extends the work of Alsubhi and colleagues [41] into specialized care contexts not previously examined. An additional important stabilizing factor mentioned by all interviewees was family‐friendly and age‐friendly shifts. Due to the demographic change in Austria [4], many older nurses have to work far beyond the prior retirement age. Promoting flexible, age‐friendly systems is therefore vital to facilitate the continued use of older workers’ expertise. Such systems not only recognize the value of experienced workers but also create an inclusive environment that encourages their continued contribution to the workforce, thereby helping to retain highly specialized professionals within the system [42, 43]. This age‐specific finding adds a new retention perspective within the contexts of LTC and ICU.

*Remuneration and Social Benefits* were perceived as stabilizing when there were additional financial incentives for taking on extra shifts, equal pay across all settings, and social benefits such as health promotion offers. Although remuneration was mentioned as a stabilizing force, interviewees emphasized that payment only contributes to a limited extent. This is also exemplified in the recent work by Chua et al. [44], who found that increasing the monetary component alone is unlikely to improve nurses′ intent to stay, thus demonstrating that retention is multifaceted. While financial incentives are consistently cited in the broader nursing retention literature [10, 45, 46], our findings reveal a setting‐specific pattern. Financial incentives for extra shifts were reported exclusively in ICU, reflecting the incentive structures typical of intraclinical environments. No comparable financial incentive for extra shifts was reported in the LTC facilities. This structural distinction between ICU and LTC remuneration practices has received limited empirical attention in the Austrian healthcare context.

*Time for Family and Proximity to Workplace* provided important insights into stabilizing off‐the‐job factors among LTC nurses. Whereas time for family was important in both settings, proximity to the workplace was a contributing factor only for nurses in LTC facilities. By contrast, critical care nurses underlined that the perceived prestige of their role supported their desire to remain in this setting. These setting‐specific results align with findings reported by Singh and colleagues [47].

*Responsibility and Ethical Challenges.* While work autonomy and supportive organizational conditions and work environments ensured the stability of the nursing workforce in both settings, bearing excessive and sole responsibility had precisely the reverse effect. Particularly when responsibility for challenging ethical decisions had to be carried alone, interviewees reported an increased desire to leave the setting. Nursing staff in LTC facilities specifically highlighted the limited availability of physicians as negatively affecting their commitment to the institution. They asserted that sharing responsibility and decision‐making within interdisciplinary teams could enhance their engagement and retention in LTC. These interpretations also align with findings from other studies showing that a less favorable perceived ethical decision‐making climate facilitates moral distress [48] as well as the desire to quit [49]. These findings also resonate with Tummers and colleagues [7], who highlighted the burden of sole responsibility in LTC as a key driver of attrition. However, the present study contributes a comparative dimension. While ethical dilemmas and moral distress were the dominant destabilizing mechanism in ICU, it was the absence of physician presence and the resulting weight of sole clinical decision‐making, leading to continuous moral distress, that emerged as the critical destabilizer in LTC. This phenomenon has similarly been documented in prior research examining the determinants of moral distress among nurses in LTC [50].

*Interpersonal Relations and Power Dynamics.* Interpersonal relational problems were reported as destabilizing factors in both settings, particularly regarding generational conflicts within the nursing or multiprofessional team. Generational conflicts within the nursing profession can also be conceptualized as power dynamic structures, primarily emerging between nurses possessing extensive professional experience and newly trained nurses entering the field. ICU nurses indicated that the pronounced hierarchical structures prevalent within the hospital sector significantly influence their experience of power dynamics in the workplace, particularly, but not exclusively, between nurses and physicians. While a trend toward flatter hierarchies was noted among interviewees, abuse of power by physicians or nursing managers still significantly contributes to withdrawal behavior among frontline nurses. This interpretation is also in line with the findings of a recent study by Bolt and colleagues [51]. Our findings demonstrate that power dynamics manifest differently across settings. In ICU, hierarchical nurse–physician dynamics were the dominant concern, while in LTC, interpersonal conflicts within the nursing team itself were more dominant. This setting‐specific expression of power dynamics adds granularity to a literature that has largely treated these phenomena as uniform across nursing contexts.

*Staffing Resources and Shift Work.* Consistent with previous research, decreased staffing levels and shift work were identified as destabilizing factors within both work environments. Notably, adverse health outcomes associated with shift work, such as depression, impaired cognitive functioning, and insomnia, were highlighted by participants as significant contributors to their decision to leave the setting. These findings are also in line with a previous study by Blytt et al. [52], which revealed that shift work disorder (SWD) serves as a catalyst for decisions to leave the workplace.

*Lack of Professional Challenge and Development* was perceived as a destabilizing force, particularly among LTC frontline nurses. Limited opportunities for professional development, along with the underutilization of nurses’ full range of professional skills, drive nurses out of LTC. Younger nurses in particular often transfer back to the hospital setting to further develop their professional careers [9]. This finding is particularly significant in the Austrian healthcare context, where the recent expansion of professional fields such as Community Nursing and Acute Community Nursing has created new exit pathways for LTC nurses seeking professional growth, a structural feature of the Austrian labor market that has not previously been examined in relation to LTC retention.

*Caring for Relatives, Professional Opportunities, Ease of Movement and Image of Nursing Profession.* Among off‐the‐job destabilizing factors, caring responsibilities for relatives placed interviewees in a particularly precarious situation. Flexible working‐time models can help nursing managers mitigate this pressure and reduce the departure of skilled nurses. However, professional opportunities, ease of occupational movement, and the broader image of the nursing profession were perceived as considerably greater challenges to workforce retention, particularly in LTC facilities. Ease of movement resulting from expanding professional opportunities (e.g., Community Nursing), the global shortage of nursing staff, and the image of the nursing profession within some Austrian care institutions were all reported to significantly decrease nurses’ retention in LTC. Nursing management should therefore invest in creating retention‐promoting work environments to stabilize the workforce, a recommendation consistent with the implications drawn by Bratt and colleagues [9].

*Work-Related Psychological Exhaustion and Trauma* were identified as significant contributing factors to nurses leaving both ICU and LTC facilities. While the severity of patients’ conditions and their personal misfortunes place a considerable burden on nursing staff in critical care, residents suffering from geriatric psychiatric illnesses represent a significant challenge for nursing staff in LTC. Greater emphasis should therefore be placed on supporting nurses in managing ethically, emotionally, or otherwise traumatic situations, particularly where they perceive themselves as being left to cope with such challenges alone. Moral distress also contributes to nurses leaving the profession, thereby significantly destabilizing the retention of frontline nurses. Particular emphasis should therefore be placed on reducing morally distressing situations by offering ethical supervision and promoting suitable coping strategies [48, 50, 53, 54].

*The COVID-19 Crisis* and the associated scarcity of resources during that period were perceived as major burdens by frontline nurses and nursing managers in both settings. Critical care nurses expressed considerable frustration regarding their ability to deliver effective care, citing increased patient volume and elevated mortality rates as contributing factors. This frustration stemmed from a perceived lack of success in their professional roles, compounded by challenges related to inadequate staffing and limited capacities. Similarly, nurses in LTC facilities reported feeling particularly burdened by insufficient regulatory frameworks instituted by management, given that a significant proportion of COVID‐19–related deaths occurred within nursing homes. This observation is consistent with the findings of Thompson and colleagues [55]. Nurses in both clinical settings reported a significant increase in workload since the onset of the pandemic, which they believe adversely affected the quality of care delivered. These findings further align with existing literature highlighting the heightened workload, compromised quality of care, and moral distress experienced by the nursing workforce during the pandemic [56].

### 4.1. Contributions to the Literature

Taken together, the findings of this study make several distinct contributions to the existing literature on nurse retention, which can be considered on both an empirical and a theoretical level.

*Empirically*, this study advances the literature in three ways. First, while the majority of prior research has examined retention factors within a single nursing setting or across the general nursing population [7, 28, 29], this study adopts an explicitly comparative design, revealing that although many stabilizing and destabilizing forces are shared across ICU and LTC, their salience, intensity, and expression differ in contextually meaningful ways. Second, this study provides the first empirical, qualitative investigation of stabilizing and destabilizing retention factors across ICU and LTC settings within the Austrian healthcare system, a context characterized by a distinct professional structure, labor market dynamics, and emerging care roles that differ from the Anglo‐American and Scandinavian settings that dominate the existing literature. Third, by incorporating the perspectives of both frontline nurses and nursing managers across multiple hierarchical levels, this study offers a more comprehensive and organizationally grounded understanding of retention dynamics than studies relying on single‐informant designs.

*Theoretically*, this study advances the understanding of nurse retention in several important ways. By applying JE theory [18], with its dual on‐the‐job and off‐the‐job structure, to specialized deviant nursing contexts, this study extends the application of JE beyond the general workforce settings in which it has most commonly been tested [12, 17], demonstrating its analytical value in high‐pressure healthcare environments. Specifically, by applying JE across two distinct specialized care settings, this study shows that the relative weight of JE’s core dimensions, fit, links, and sacrifices, is not uniform but varies meaningfully by context. This suggests that JE should be understood not as a context‐neutral framework, but as one whose dimensional priorities are shaped by the structural and cultural characteristics of the specific work environment, a theoretical refinement with implications for how JE is applied and measured in future healthcare research. The comparative design further reveals that the on‐the‐job/off‐the‐job distinction within JE itself operates differently across settings. In critical care, on‐the‐job factors dominated both the stabilizing and destabilizing landscape, whereas in LTC, off‐the‐job forces, such as caring responsibilities for relatives, professional image, and ease of movement played a comparatively larger role in retention decisions. Future theoretical refinements of JE in nursing contexts may therefore benefit from incorporating setting‐specific weighting of the on‐the‐job and off‐the‐job dimensions, rather than treating them as equally applicable across all care environments. Finally, this study contributes to ongoing theoretical debates about the relationship between job satisfaction and retention. Consistent with Lee and colleagues’ argument [17] that job satisfaction alone cannot account for retention behavior, the findings show that nurses in both settings remained in their positions despite reporting significant destabilizing pressures, because embeddedness forces operated simultaneously to bind them to their roles. This empirically reinforces JE’s theoretical claim that retention reflects not merely the absence of dissatisfaction, but the presence of positive embedding forces.

Overall, these empirical and theoretical insights call for a more contextually sensitive application of JE theory in healthcare workforce research and support the broader argument that retention theories developed in general organizational settings require empirical validation and contextual adaptation before being applied to specialized, high‐pressure professional healthcare environments.

### 4.2. Limitations and Future Research

Although this study has numerous strengths, it also presents certain limitations, primarily relating to its methodological approach. Data gathered through interviews and focus group discussions may be subject to social desirability bias [32], warranting cautious interpretation of the findings. While efforts were made to ensure the trustworthiness of the study, the findings remain bound to the specific public healthcare contexts investigated and cannot be generalized beyond them. Future research should therefore replicate and extend this comparative design across a broader range of settings and healthcare systems, including private institutions as well as both inner and outer clinical environments, and consider applying alternative methodological approaches to strengthen generalizability.

## 5. Conclusion

This comparative case study shows that nurse retention cannot be addressed through generic, one‐size‐fits‐all strategies. While ICU and LTC nurses share a common core of stabilizing and destabilizing forces, the weight and expression of these forces diverge in ways that matter for practice: ICU retention hinges more on managing on‐the‐job ethical strain within cohesive teams, while LTC retention depends more on redistributing clinical responsibility and strengthening the profession’s standing off‐the‐job. Recognizing this divergence, rather than treating nursing as a homogeneous workforce, is a necessary first step toward retention policies that actually fit the setting they are meant to serve.

### 5.1. Implications for Nursing Management

Findings point to concrete, setting‐specific levers rather than one‐size‐fits‐all retention policy. In ICU, managers should prioritize structured psychological and ethical support (e.g., regular debriefing or ethics consultation) to address moral distress from sole responsibility in high‐acuity decisions and should preserve collaborative team routines, since cohesion here is tied directly to patient safety rather than general job satisfaction. Financial incentives for extra shifts should be maintained but not treated as a primary retention lever.

In LTC, nursing management should focus on redistributing clinical responsibility, particularly through better integration of physician availability, to relieve nurses of isolated ethical and clinical burden. Career development pathways should be created within LTC facilities itself to prevent skilled nurses exiting to hospital settings or Community Nursing, and institutional reputation should be actively managed to compete for staff.

Across both settings, nursing managers should

• protect and expand work autonomy and participative decision‐making, since these directly enable the high‐quality care nurses are motivated to deliver.
• reduce missed nursing care by outsourcing non‐nursing tasks, freeing clinical capacity.
• implement flexible, age‐friendly scheduling to retain experienced older staff and support employees with caregiving responsibilities.
• address generational and hierarchical power dynamics through flatter structures and an open error culture.
• build systematic emotional‐support structures (e.g., ethical supervision, coping‐strategy training), rather than relying on individual resilience.

These measures should be implemented as differentiated, context‐sensitive bundles rather than uniform policy, given that the salience of each factor diverges meaningfully between ICU and LTC.

**Nomenclature**
ICU: Intensive care unit/ critical care units
LTC: Long‐term care
NM: Nursing managers
FN: Frontline nurses
JE: Job embeddedness

## Author Contributions

Tanja Lesnik contributed to data collection, data analysis, data synthesis, and the draft of the manuscript.Olivia Kada contributed to data analysis, data synthesis, and read and approved the final manuscript.

## Funding

This research received no specific grant from any funding agency in the public, commercial, or not‐for‐profit sectors. Open access funding was provided by Universitat Klagenfurt/KEMÖ.

## Ethics Statement

This study was approved by the ethical committee of the University of Klagenfurt (Ethical Approval No.: 2023‐053). Informed consent was obtained from all study participants and the institutions in which they work.

## Consent

Please see Ethics Statment.

## Conflicts of Interest

The authors declare no conflicts of interest.

## Supporting Information

Additional supporting information can be found online in the Supporting Information section.

## Supporting information


**Supporting Information** The Supporting Information attached to this manuscript comprises Additional Files 1 to 5. Additional File 1 outlines the purposive sampling plan, detailing the selection of interview participants and the selected cases, which include public hospitals and public nursing homes. The file also includes the research questions, thereby elucidating the objectives of the study. Additional File 2, the coding guideline, explains the coding structure applied to gather the results of this study. It includes main categories and subcategories, along with anchor examples and frequencies of occurrence, allowing for multiple citations per participant. Additional File 3 presents the interview questions employed in this study. The semistructured interview guide served as a framework to guide participants throughout the interview process. This interview guide encompassed inquiries directed toward both frontline nurses and nursing managers. Among the questions included were, for instance, the following: (1) what is the main reason why you feel comfortable in your field as a nurse and want to continue working there? (2) Do you feel that you can realize your potential in your current area and make good use of your strengths? (3) In your experience, what are the reasons why qualified nursing staff have left the intensive care sector voluntarily (not because of pregnancy, retirement, or illness)? (4) How can you keep employees in the intensive/LTC sector for a long time and, above all, keep them productive? Additional File 4 encompasses the COREQ checklist, which systematically documents all items pertinent to this qualitative study. This checklist enhances the study’s transparency and comprehensiveness, ensuring that the reporting standards are rigorously adhered to. Additional File 5 presents the criteria for trustworthiness by Guba and Lincoln, encompassing key criteria such as credibility, transferability, confirmability, and dependability. This additional file also outlines the applied strategies in this study to achieve adherence to these criteria, thereby enhancing the rigor of the research findings.

## Data Availability

The dataset includes personal information, thereby necessitating restrictions on its dissemination. The researchers are committed to upholding the integrity of the data as promised.
