# Supplementary material for: Stabilizing and Destabilizing Factors of Nurse Retention: A Comparative Analysis of Two Healthcare Settings in Austria
Source: J Nurs Manag. 2026 Aug 7;2026:8957256. doi: 10.1155/jonm/8957256 (PMC13451637; doi:10.1155/jonm/8957256)
Supplement: Supplementary file 1 [file JONM-2026-8957256-s001.zip › Additional file 1_Purposive Sampling Plan.docx]

**Purposive Sampling and Sampling Plan**

Select respondents (Registered Nurses and Nursing Managers from public Critical Care & Long-term Care facilities in Austria)

Initial contact
with Nursing director (Gatekeeper) and subsequent initial contact with participants

Informed Consent -> Conduct interviews & focus group discussions and taking notes

**YES**

**NO**

Data Coding and categorisation, theme development

Transcribe and evaluate

Analyse

Search for Alternative

**Select additional participants**

**RQ1:** What are the key on-the-job and off-the-job stabilizing and destabilizing factors that influence critical care and long-term care nurses’ decision to remain in their work environment?

**RQ2:** How do stabilizing and destabilizing factors vary across these deviant settings, and what are the underlying similarities and differences?

| **Setting  (Case)** | **Sample** | **Inclusion criteria** | **Exclusion criteria** | **n  Participants** | **n  Institutions** | **Level of Nursing Management** | **Level of nurses** |
| --- | --- | --- | --- | --- | --- | --- | --- |
| **Critical Care** | **A (RN)** | Registered Critical Care Nurses (RN) with min. 1 year of professional experience in critical care in public hospitals in Austria | Nursing Aids, other healthcare professionals, Medical Doctors from normal wards in hospital setting, RN with less than 1 year of professional experience in critical care | Minimum 10 -> depends on theoretical saturation (Denzin & Lincoln, 2018)  In total: 16 | 9 |  | Registered Nurses (RN) |
|  | **B (NM)** | Nursing Managers (NM) of lower, middle and upper management levels in public hospitals in Austria | Nursing Managers (NM) of private hospitals, or outer clinical facilities | Minimum 10 -> depends on theoretical saturation (Denzin & Lincoln, 2018)  In total: 16 | 9 | All levels -> NM of lower, middle and upper nursing management |  |
| **Long-term Care** | **C (RN)** | Nursing Managers (NM) of lower, middle and upper management levels in public nursing homes in Austria | Nursing Managers (NM) of private nursing homes, or public/private hospitals | Minimum 10 -> depends on theoretical saturation (Denzin & Lincoln, 2018)  In total:10 | 10 | All levels -> NM of lower, middle and upper nursing management |  |
|  | **D (NM)** | Registered Nurses (RN) with min. 1 year of professional experience in long-term care in public nursing homes in Austria | Nursing Aids, other healthcare professionals, Medical Doctors, Healthcare professionals from hospital setting, RN with less than 1 year of professional experience in long-term care | Minimum 10 -> depends on theoretical saturation (Denzin & Lincoln, 2018)  In total: 10 | 10 |  | Registered Nurses (RN) |

**Cases**

| **Cases** | **Type of hospital** | **# Employees (interdisciplinary)** | **#outpatient patients per year** | **# inpatient patients per year** | **Hospital beds** |
| --- | --- | --- | --- | --- | --- |
| **Public Hospitals** |  |  |  |  |  |
| A | General hospital | 350 | 18,700 | 8,000 | 140 |
| B | General hospital | 2,041 | 213,526 | 27,720 | 619 |
| C | General/ University hospital | 8,846 | 538,478 | 62,174 | 1,922 |
| D | General/ University hospital | 7,469 | 883,127 | 341,225 | 1,282 |
| E | General/ University hospital | 4,226 | 888,484 | 58,501 | 1,040 |
| F | General hospital | 550 | 50,000 | 12,500 | 196 |
| G | Specialty hospital | 348 | 852 | 1,369 | 150 |
| H | Specialty hospital | 314 | 45,260 | 5,056 | 100 |
| I | General hospital | 4,836 | 624,783 | 55,078 | 1,131 |
| **Public Nursing Homes** | **# Employees (interdisciplinary)** | **Long-term care beds** | **Transition care (beds)** | **Short-term care (beds)** | **Day Care** |
| J | 81 | 102 |  |  | X |
| K | 52 | 75 |  |  | X |
| L | 85 | 79 | X | X | X |
| M | 125 | 95 |  |  |  |
| N | 55 | 75 | X | X | X |
| O | 124 | 122 | X | X | X |
| P | 262 | 215 |  | X | X |
| *Q | 4,800 | 8,000 | X | X | X |

*Merger of multiple Nursing Homes

References

Denzin, N., & Lincoln, Y. (2018). *The SAGE Handbook of Qualitative Research: Fifth Edition*. SAGE Publications.
