# Supplementary material for: Stabilizing and Destabilizing Factors of Nurse Retention: A Comparative Analysis of Two Healthcare Settings in Austria
Source: J Nurs Manag. 2026 Aug 7;2026:8957256. doi: 10.1155/jonm/8957256 (PMC13451637; doi:10.1155/jonm/8957256)
Supplement: Supplementary file 1 [file JONM-2026-8957256-s001.zip › Additional file 2_Coding guideline.docx]

| **Variable** | **Category** | **Sub-category** | **Category  definition** | **Coding  rule** | **Anchor  examples** | **N***  **ICU** | **N***  **LTC** | **N***  **TOTAL** |
| --- | --- | --- | --- | --- | --- | --- | --- | --- |
| SOnF_ Stabilizing on-the-job factors | 1 Stabilizing on-the-job factors | 1.1 Empowering leadership & positive team culture | Stabilizing job-related factors, including empowering leadership or positive team culture, as contributing factors to retention among frontline nurses in long-term care (LTC) and critical care (ICU) work environments. These endogen variables stabilize or also enhance retention among this workforce. | Only text material that contains stabilising mechanisms in the area of empowering leadership or positive team culture. | *“I do think that the manager should take time for the employees and that everyone is listened to and talked to and asked how they are doing and that the employee simply feels comfortable and at ease in the team and that they don't have to be afraid if they ask the management something. That they can simply talk about it openly. The feeling of well-being and communication and valuable interaction. (ICU_nurse_4, Pos. 346)”* | *53* | *29* | *46* |
|  |  | 1.2 Organisational conditions & work environment | Stabilizing job-related factors, including organisational conditions and work environment, as contributing factors to retention among frontline nurses in long-term care (LTC) and critical care (ICU) work environments. These endogen variables stabilize or also enhance retention among this workforce. | Only text material that contains stabilising mechanisms in the area of organisational conditions and work environment. | *“Yes, and for the older generations, of which I am one, I would like us to be able to take advantage of programmes such as partial retirement when we get older. Because I have to be honest, you realise that after a certain time you're no longer as productive as you used to be. If this is encouraged, there will also be fewer sick days, and more satisfaction. And perhaps older people can work longer and still do well in a company. That's what I want. I would also like to be able to continue working there. (Interview 9_nurse_LTC_1, Pos. 9)”* | *116* | *59* | *175* |
|  |  | 1.3 Remuneration & social benefits | Stabilizing job-related factors, including remuneration and social benefits,  as contributing factors to retention among frontline nurses in long-term care (LTC) and critical care (ICU) work environments. These endogen variables stabilize or also enhance retention among this workforce. | Only text material that contains stabilising  mechanisms in the area of remuneration and social benefits. | *“Yes, I have to say that in the beginning, being a nurse was very attractive from a financial point of view.*  *But I think it's a vocation anyway, so you can't just do it for the money.*  *Therefore, money is not the best motivator. (Interview 4_nurse_LTC_4, Pos. 1)”* | *67* | *39* | *106* |
| SOfF_ Stabilizing off-the-job factors | 2 Stabilizing off-the-job factors | 2.1 Time for family & Proximity to workplace | Stabilizing non-job-related factors, including time for family or proximity to workplace, as contributing factors to retention among frontline nurses in long-term care (LTC) and critical care (ICU) work environments. These exogen variables stabilize or also enhance retention among this workforce. | Only text material that contains stabilising mechanisms in the area of time for family or proximity to workplace. | *“I believe that it is becoming increasingly important for them to reconcile work and family life. I know this because we have just completed a project on this topic and conducted a survey (Interview 4_NM_ICU, Pos. 7)”* | *24* | *17* | *41* |
|  |  | 2.2 Prestige | Stabilizing non-job-related factors, including prestige, as contributing factor to retention among frontline nurses in long-term care (LTC) and critical care (ICU) work environments. These exogen variables stabilize or also enhance retention among this workforce. | Only text material that contains stabilising mechanisms in the area of prestige. | *“One of the reasons why I like working in intensive care and want to stay there is the ‘reputation’ or that it is a profession or job that you can be proud of and, above all, that it is very highly regarded in society. When you say that you are an intensive care nurse, everyone is always amazed. (Focus group_nurse__ICU_1_2, Pos.31)“* | *4* |  | *4* |
| DSOnF_ Destabilizing on-the-job factors | 3 Destabilizing on-the-job factors | 3.1 Responsibility & Ethical challenges | Destabilizing job-related factors among frontline nurses that include responsibility or ethical challenges as contributing factors to their decision to quit long-term care (LTC) or critical care (ICU) work environments. These endogen variables destabilize or also enhance the decision to leave the setting. | Only text material that contains destabilising mechanisms in the area of responsibility or ethical challenges. | *“From my experience in the long-term care sector, I think one main reason is the big responsibility that the qualified nurses in a nursing home have to bear and certainly the fact that we don't have a doctor on site 24 hours a day. So, we don't have a doctor in the institution around the clock, and we have a lot of responsibility and have to make a lot of decisions, so that's certainly one of the main reasons, I would say, because we have a lot of responsibility and have to make a lot of decisions on ourselves. (Interview 5_NM_LTC, Pos. 1)”* | *27* | *23* | *50* |
|  |  | 3.2 Interpersonal relations & Power dynamics | Destabilizing job-related factors among frontline nurses that include interpersonal relations or power dynamics as contributing factors to their decision to quit long-term care (LTC) or critical care (ICU) work environments. These endogen variables destabilize or also enhance the decision to leave the setting. | Only text material that contains destabilising mechanisms in the area of interpersonal relations or power dynamics. | *“In which case would I leave? If this team constellation gets completely out of hand and it's no longer possible for me to work here. If there is bullying, if the boss is no longer behind me, if there is no open ear for the problems and needs of the employees. Or if the staffing situation gets even worse and I have the feeling that this is such an emergency situation that I can't be part of it. I can no longer be a part of this team. (Interview 6_nurse_LTC_6, Pos. 7)”* | *89* | *12* | *101* |
|  |  | 3.3 Staffing resources & Shift work | Destabilizing job-related factors among frontline nurses that include staffing resources or shift work as contributing factors to their decision to quit long-term care (LTC) or critical care (ICU) work environments. These endogen variables destabilize or also enhance the decision to leave the setting. | Only text material that contains destabilising mechanisms in the area of staffing resources or shift work. | *“A few years ago, they just ran out of staff in one residential area, there was a trainee and the cleaner who managed the whole ward and there wasn't a qualified nurse or a proper nursing assistant on duty. There was just no other option, there was nobody else. That's very problematic. (Interview 1_nurse_LTC_1, Pos. 6)*” | *10* | *29* | *39* |
|  |  | 3.4 Lack of professional challenge & Professional development | Destabilizing job-related factors among frontline nurses that include lack of professional challenge or professional development as contributing factors to their decision to quit long-term care (LTC) or critical care (ICU) work environments. These endogen variables destabilize or also enhance the decision to leave the setting. | Only text material that contains destabilising mechanisms in the area of lack of professional challenge or professional development. | *“There is no further professional development, there is nothing. Because the residents have to be cared for, how am I supposed to professionally develop further? There may be a course in aroma care that I can do, but we already have that, it's standard. (Interview4_NM_LTC, Pos. 3)”* |  | *11* | *11* |
|  |  | 3.5 Work related psychological exhaustion & Trauma | Destabilizing job-related factors among frontline nurses that include work related psychological exhaustion or trauma as contributing factors to their decision to quit long-term care (LTC) or critical care (ICU) work environments. These endogen variables destabilize or also enhance the decision to leave the setting. | Only text material that contains destabilising mechanisms in the area of work related psychological exhaustion or trauma. | *“Then we often have geriatric psychiatric cases where it's not the body that needs to be treated, but the person's own psyche. Then you really have to treat your own psyche, which is extremely challenging. (Interview 2_NM_LTC, Pos. 2)”* | *11* | *5* | *16* |
| DSOfF_ Destabilizing off-the-job factors | 4 Destabilizing off-the-job factors | 4.1 Caring for relatives | Destabilizing non-job-related factors among frontline nurses that include caring for relatives as contributing factors to their decision to quit long-term care (LTC) or critical care (ICU) work environments. These exogen variables destabilize or also enhance the decision to leave the setting. | Only text material that contains destabilising mechanisms in the area of caring for relatives. | *“And we also have children or parents who need professional help because of an illness or whatever, so everyone has to take responsibility in their family too. (Focus group_nurse_ICU_3_2, Pos. 218)”* | *4* |  | *4* |
|  |  | 4.2 Professional opportunities, ease of movement & Image of nursing profession | Destabilizing non-job-related factors among frontline nurses that include professional opportunities, ease of movement or image of nursing profession as contributing factors to their decision to quit long-term care (LTC) or critical care (ICU) work environments. These exogen variables destabilize or also enhance the decision to leave the setting. | Only text material that contains destabilising mechanisms in the area professional opportunities, ease of movement or image of nursing profession. | *“For example, we now also have cases where staff have specifically moved away from intensive care, for example to the preclinical emergency field. We have a completely new profession elsewhere, this Acute Community Nurse (CAN), where intensive care staff suddenly switch to an extramural area. This is a particularly interesting project and didn't exist in the past. Today nurses are more flexible. (Interview 11_NM_ICU, Pos. 13)”* | *16* | *11* | *27* |
|  |  | 4.3 COVID-19 crisis | Destabilizing non-job-related factors among frontline nurses that include the COVID-19 crisis as contributing factors to their decision to quit long-term care (LTC) or critical care (ICU) work environments. These exogen variables destabilize or also enhance the decision to leave the setting. | Only text material that contains destabilising mechanisms in the area of the COVID-19 crisis. | *“Yes, in recent years, especially after COVID-19, it has actually become more and more difficult. Before, before COVID-19, it was actually good to work, but during and after COVID-19 I really have to say that there has been a rupture in some way, I think the workload in long-term care is extremely high since then. (Interview 7_nurse_LTC_7, Pos. 1) “* | *3* | *3* | *6* |

*Note: The interviews were conducted in German, with guidelines and interviewee quotes translated into English.*

**Multiple citations possible*
