# Supplementary material for: Stabilizing and Destabilizing Factors of Nurse Retention: A Comparative Analysis of Two Healthcare Settings in Austria
Source: J Nurs Manag. 2026 Aug 7;2026:8957256. doi: 10.1155/jonm/8957256 (PMC13451637; doi:10.1155/jonm/8957256)
Supplement: Supplementary file 1 [file JONM-2026-8957256-s001.zip › Additional file 3_Interview Guide.docx]

**Interview Guide for Frontline Nurses (ICU/LTC)**

**Introductory** **question**

1. Why did you decide to enter the long-term care sector as a nurse?

**REASONS, INFLUENCING FACTORS AND NEEDS**

2. What is the main reason why you feel comfortable in your field as a nurse and want to continue working there?

a. How did your needs change as your career progressed?

b. What was important to you for working well in your field in the early years of your career and what is important to you today?

3. Have your strengths and competences changed since you started your career? Do you feel that you will be able to utilise your strengths well in your field in the future?

a. If so, what is beneficial for you?

b. If not, what prevents you from doing so?

c. What do you need to ensure that this continues to be possible?

4. Do you feel that you can realise your potential in your current area and make good use of your strengths? Where do you experience this - can you give me an example?

a. Can you imagine that this will remain the case in the coming years or do you expect it to change for you? Please describe.

b. Do you notice any restrictions in terms of professional development in your field as you get older? Why? Why not?

5. On the one hand, there is much discussion in the media that the length of time carers stay in nursing is short and that carers often leave the profession voluntarily. On the other hand, employees are getting older and are approaching retirement. What is the specific situation in your ward/area/in your organisation? How do you see the problem for the care sector in which you work? In your opinion, is there a need for action here?

6. How can you as a nurse be kept in your job as a long-term carer for a long time? What do you need so that you can develop optimally? Give examples.

a. What are the supporting and driving forces behind your decision to stay in your field?

b. What hinders your decision to stay in your field?

c. What do you need to optimise your professional development?

7. How are the expertise and many years of experience of ‘older’ employees retained in your area? Is this valued? Tell us.

***FINAL QUESTION***

8. If you were a ‘magician’ for a day and could do anything you wanted without taking resources into consideration, what measures would you implement to ensure that nurses continue to work in the long-term care sector?

**Interview Guide for Nursing Managers (ICU/LTC)**

**Introductory** **question**
1. In your experience, what reasons motivate employees to enter the intensive care or long-term care sector?

**REASONS, INFLUENCING FACTORS AND NEEDS**

2. In your experience, what are the reasons why qualified nursing staff have left the intensive care sector voluntarily (not because of pregnancy, retirement or illness)? Give some examples.

3. Conversely, what is the main reason why employees feel comfortable in the field of intensive care or long-term care and want to stay there in the long term?

a. How do these needs change with increasing years in the profession? Do they change at all? If yes, how? If not, why not? Can you give me an example?

b. What do you think is important to younger employees?

c. What do you think is important for more experienced employees?

4. How do the strengths and potentials of employees change over the years?

a. Do you recognise different strengths and potential in older employees?

b. Are there activities/tasks for which older employees are particularly suited? Give examples.

c. Do you see any restrictions on professional development in your area after a certain age? Why? Why not?

d. How do you see the professional development opportunities for older employees?

5. On the one hand, there is much discussion in the media that the length of time carers stay in the care sector is short and that carers often leave the profession voluntarily. On the other hand, employees are getting older and are approaching retirement. What is the specific situation in your area? How do you see the problem in your area? Is there a need for action here?

6. How can you keep employees in the intensive/long-term care sector for a long time and, above all, keep them productive?

a. What is your role as a manager in this?

b. What are you already implementing and what are you planning for the future?

c. Do you pursue an overarching strategy in the area of employee retention or what does this look like for you specifically?

d) What makes it easier or more difficult for you to implement your measures?

7. What is being done to retain and optimally utilise the expertise of experienced employees?

8. How can you keep so-called ‘high performers’ in the department? What do you think is necessary to retain these employees?

9. Can you as a manager support your employees in their professional development? If yes, how do you do this? If not, why is there nothing you can do?

10. When you think of employee retention measures, which would be easy and inexpensive to implement?

a. Which measures would involve a lot of effort and high costs?

***FINAL QUESTION***

11. If you were a ‘magician’ for a day and could do anything you wanted without taking resources into account, what measures would you implement to keep your nursing staff in the profession?

*Notes:*

….

**Theory for establishment of interview questions**

Holtom, B.C. & O'Neill, B.S. (2004). Job Embeddedness: A Theoretical Foundation for Developing a Comprehensive Nurse Retention Plan. *The Journal of Nursing Administration*, *34*(5), 216–227.

Lee, T., Hom, P., Eberly, M., & Junchao, L. & Mitchell, T. (2017). On the next decade of research in voluntary employee turnover. *Academy of Management Perspectives*, *31*(3), 201–221.

Lee, T. W., & Burch, T. C. & Mitchell, T.R. (2014). The Story of Why We Stay: A Review of Job Embeddedness. *Annual Review of Organizational Psychology and Organizational Behavior* (1), 199–216.

Mitchell, T. R., Holtom, B. C., Lee, T. W., & Sablynski, C.J. & Erez, M. (2001). Why People Stay: Using Job Embeddedness to Predict Voluntary Turnover, *44*(6), 1102–1121.
