# Supplementary material for: Stabilizing and Destabilizing Factors of Nurse Retention: A Comparative Analysis of Two Healthcare Settings in Austria
Source: J Nurs Manag. 2026 Aug 7;2026:8957256. doi: 10.1155/jonm/8957256 (PMC13451637; doi:10.1155/jonm/8957256)
Supplement: Supplementary file 1 [file JONM-2026-8957256-s001.zip › Additional file 5_Guba and Lincolns criteria.docx]

| **Criteria** | **Purpose** | **Original strategies** | **Strategies applied in this qualitative study** |
| --- | --- | --- | --- |
| **Credibility** | To ensure that the research process and findings were done with integrity and confidence in the truth of the findings (not only from the researcher’s point of view) | - Prolonged engagement - Persistent observation - Triangulation - Member checking - Peer debriefing | - Prolonged engagement (Authors dedicated 5 months to data collection in the field) - Persistent observations (Authors focused on the most relevant factors) - Triangulation (Authors triangulated perceptions of nurses with perceptions of nursing managers in critical care and long-term care) - Peer debriefing (Authors engaged with each other to discuss assumptions and interpretations through the coding process) |
| **Transferability** | To show that findings from the study can be transferred to other contexts thereby not aiming for quantitative generalizability. | - Thick description of setting, participants, research process - Purposive sampling | - Thick description (Authors described the research contexts, sampling method, participants characteristics and data analysis process) - Purposive sampling (Authors selected participants with the help of gatekeepers and snowballing technique to collect the most knowledgeable agents) |
| **Confirmability** | To show that the findings are clearly derived from the data and not from the researcher’s predispositions. | - Reflexivity - Audit trail - Confirmability audit | - Reflexivity (Author 1, who is close to the settings, maintains awareness of their biases, values and influence on the research and therefore engaged in deep peer debriefing sessions with Author, who has no relation to the settings) - Audit trail (Authors put every effort to create the research process intersubjective comprehensible as this is an important criterion of the Content analysis of Mayring (2022). |
| **Dependability** | To ensure that if the study were repeated in the same context, with the same methods and participants, similar results would be obtained. | - Audit trail - Code-recode strategy - Peer examination - Stepwise replication | - Audit trail (Authors put every effort in keeping detailed records of the research process using the COREQ checklist) - Peer examination (Author 1 and 2 discussed the data analysis and synthesis process several times) - Stepwise replication (Author 1 and 2 independently coded the data) |

(adapted based on Guba and Lincoln, 1989; Enworo, 2023)

**References:**

Guba, E. & Lincoln, Y. (1989). Fourth generation evaluation. Sage Publication: Newbury Park, California.

Enworo, O.C. (2023). Application of Guba and Lincoln’s parallel criteria to assess trustworthiness of qualitative research on indigenous social protection systems. *Qualitative Research Journal*. 23 (4): 372-384.

Mayring, P. (2022). *Qualitative Inhaltsanalyse: Grundlagen und Techniken* (13., überarbeitete Auflage) [Print]. Beltz Verlag.
